# The National Association of Dental Examiners

**Published:** 1890-11

**Authors:** J. H. Martindale

**Affiliations:** Minneapolis, Minn.


### PROCEEDINGS.

### The National Association of Dental Examiners.

The National Association of Dental Examiners held its annual session at the Elms Hotel, Excelsior Springs, Mo., Monday, Aug. 4th, 1890, at 5 p. m., President Waters in the chair. The following State Boards of Dental Examiners were represented: Illinois, by Dr. C. R. E. Koch; Iowa, by Drs. S. A. Garber, E. E. Hughes and E. D. Brower; Pennsylvania, by Dr. Louis Jack; Maryland, by Dr. T. S. Waters; Kansas, by Dr. L. C. Wasson; Ohio, by Drs. J. Taft and H. A. Smith; Minnesota, by Dr. J. H. Martindale. In consequence of the absence of Sec'y F. A. Levy (which a letter from him stated was caused by illness) Dr. Martindale was elected to serve as Secretary pro. tem. for the meeting. Minutes of the proceedings of the meeting of Aug., 1889, were then read as they appeared in the records sent on by Sec'y Levy and the same were approved. After an informal discussion upon the subject of the scope and duties of the National Association of Boards of Dental Examiners, and the relation existing between the various Boards of Examiners and the colleges. Moved by Dr. Taft, and carried, that the chair appoint three members as a committee on order of business to arrange an order of subjects to be presented before the Association for its consideration at its next session. President Waters appointed as such committee, Drs. Taft, Jack and Waters. Meeting then adjourned to sit again at 8 p. m. same day.

EVENING, AUG. 4th, 1890, 8 P. M.—Meeting called to order, Prest. Waters in the chair, roll called and minutes of previous meeting read and approved. The report of committee on order of business was called for, which consisted in the calling the attention of the Association to the following:

1st. Shall students during their term of pupilage be authorized by a State Board of Dental Examiners to temporarily practice dentistry?

2nd. What are the duties of the dental profession, and how shall their assistance be secured in maintaining the laws now in operation regulating the practice of dentistry, especial reference being had to such cases as that of the New Hampshire law now being considered in the U. S. Supreme Court?

3rd. To secure the best good to the public, by what means should the examining boards be created? By the election from societies or by appointment of Governor?

4th. What shall be the character of the examinations to be conducted by the various Examining Boards, and how far may these examinations be made uniform in character? Shall they be oral and written with also practical demonstrations of capacity?

Motion was made that the sections of said report be considered in serial order, and so acted upon—carried. Dr. Jack presented the following:

*Resolved*, That this body recommend the various Examining Boards under no circumstances to grant temporary licenses to dental students at any period during their course of instruction. To which Dr. Koch moved as an amendment the addition of the words, "whenever their State laws will permit them so to do." The resolution as amended was adopted. The association then adjourned to meet next day at 9 a. m.

Aug. 5th, 1890, 9 a. m.—The Association was called to order by Pres. Waters, roll was called and the minutes of the previous meeting read and approved. The discussion of subjects presented by the business committee at last meeting was resumed and the following was moved by Dr. Koch and carried, "That the secretary call the attention of the American Dental Association to the fact that a case involving the constitutionality of the New Hampshire dental law is now pending in the U. S. Supreme Court and that the National Association of Dental Examiners respectfully memorialize them to see to it that the case is not allowed to go by default."

Moved also by Dr. Koch and carried, "That a committee of three (3) of which Dr. Jack shall be one be appointed for the purpose of formulating desirable features of a dental law to be recommended for inclusion in the laws of the various States."

The chair appointed as such committee, Drs. Louis Jack, S. A. Garber and P. T. Smith. President Waters also appointed Drs. Wasson and Jack to fill the vacancies in the committee "upon list of reputable colleges." The following letter from Dr. L. D. Shepard, of Boston, was then read:

Dr. F. A. Levy, Sec'y Nat. Ass. Dent. Ex.:

*Dear Sir:*—At this late day, having spent considerable time in preparing a report to be presented to the other members of the committee upon the reputable colleges, I am placed by the examination just held in Mass. in a position where, if I report at all, I must exclude some of the colleges which were favorably reported upon and included in the list of last year. They are also old institutions. In an examination last week, after a careful and thorough test, we had to reject men who had been graduated from the Philadelphia Dental College, the University of Pennsylvania and other schools. I could not expect that the others of the committee would agree with me or accept my conclusions and I do not care to present a minority report. In fact, I have no place upon such a committee, as under our law, the question of the standing of a college has no weight with the board, hence I should not be a judge or arbiter of the standing of colleges. I am sorry that I did not see the question in this light till since the recent examinations, otherwise I should earlier have done what I do now—resign from the committee. I will thank you to send notice of this resignation to the other members and take such action about the report as may seem to you wise and proper. I can still take part in the Association of Dental Examiners and shall hope to be present with you.

Yours Truly, L. D. Shepard.

Boston, July 6th, 1890.

Consideration of this resignation was postponed until next meeting and the Association adjourned until 5 p. m. same day.

Meeting called to order 5 p. m. Aug. 5th, 1890. President Waters in the chair, roll called, minutes of the previous meeting read and approved. Upon motion of Dr. Taft, the resignation of Dr. L. D. Shepard from the committee on list of reputable colleges, was accepted and the secretary instructed to engross the contents of his letter of resignation upon the record book of the Association. It was suggested by Dr. Koch that inasmuch as the experiences of many of the boards of examiners, whose laws required them to exact examination of college graduates, was developing the fact that many of the graduates recently granted their diplomas from colleges included in the list of reputable colleges of this Association, were by said examinations conclusively proven to be incapable of assuming the responsibilities of dental practice, that it would be only justice to the people of the United States and kindness to the derelict colleges to inform them by a communication to the Association of Dental Faculties, now in session, that these things could not repeatedly occur without making it necessary for the National Association of Dental Examiners to cancel the names of such colleges from their list of reputable colleges. After considerable discussion upon Dr. Koch's proposition, he (Dr. Koch) was instructed to draft his remarks in the shape of a resolution and propose the same to the Association for adoption at some subsequent time.

The committee on reputable colleges then reported with favor the following list of colleges, which was adopted:

American College of Dental Surgery, Chicago.
Baltimore College of Dental Surgery, Baltimore, Md.
Boston Dental College, Boston, Mass.
Chicago College of Dental Surgery, Chicago.
College of Dentistry, Department of Medicine, University of Minnesota, Minneapolis, Minn.
Dental Department Columbian University, Washington, D. C.
Dental Department of Northwestern University, (now the University Dental College), Chicago, Ill.
Dental Department of Southern Medical College, Atlanta, Ga.
Dental Department of University of Tennessee, Nashville, Tenn.
Harvard University, Dental Department, Cambridge, Mass.
Indiana Dental College, Indianapolis, Ind.
Kansas City Dental College, Kansas City, Mo.
Louisville College of Dentistry, Louisville, Ky.
Minnesota Hospital College, Dental Department, Minneapolis, Minn. (Defunct.)

Missouri Dental College, St. Louis, Mo.
New York College of Dentistry, New York City.
Ohio College of Dental Surgery, Cincinnati, Ohio.
Pennsylvania College of Dental Surgery, Philadelphia, Penn.
Philadelphia Dental College, Philadelphia, Pa.
School of Dentistry of Meharry, Medical Department of Central Tennessee College, Nashville, Tenn.
St. Paul Medical College, Dental Department, St. Paul, Minn. (Defunct.)
University of California, Dental Department, San Francisco, California.
*Northwestern College of Dental Surgery, Chicago. (Defunct)
University of Iowa, Dental Department, Iowa City, Ia.
University of Maryland, Dental Department, Baltimore, Md.
University of Michigan, Dental Department, Ann Arbor, Mich.
University of Pennsylvania, Dental Department, Philadelphia, Pennsylvania.
Vanderbilt University, Dental Department, Nashville, Tenn.

Dr. Wasson moved that the secretary be instructed to send a printed copy of this report to all the deans of the dental colleges in the United States and Canada, also to the boards of dental examiners of all States having dental laws, so ordered.

The committee upon formulating a scheme for ensuring uniformity in the State laws regulating the practice of dental surgery, reported through their chairman, Dr. Louis Jack, as follows:

Mr. President of the National Association of Dental Examiners your committee appointed by the resolution of Dr. Koch, "for the purpose of formulating desirable features of a dental law, to be recommended for incorporation in the laws of the various States" have met, and would present to you their consideration of several propositions.

We understand the purpose of the above resolution to be an endeavor to secure as far as may be possible a state of uniformity

* The diplomas of this college are discredited after 1889.

in the laws of the different States, with the view that as new laws may be made and old ones amended, at length the above stated condition of uniformity may be attained. These propositions are:

1st. That there shall be created Boards of Examiners or Censors, in each State.

2nd. That these Boards be formed by election carefully conducted in each of the State dental societies.

3rd. That ten years continuous practice at the time of passage of the law, shall qualify for the continuance in practice.

4th. That said boards shall have power to conduct examinations and grant certificates to those not graduates, providing the candidates present satisfactory certificates or affidavit of having had at least five calender years of instruction.

5th. These, and all examinations, shall be both oral and written and the candidate shall also be subjected to tests of practical skill.

6th. We believe that the Boards should have power to examine graduates of dentistry.

7th. We do not consider that medical graduates without special qualification should be permitted to practice dentistry.

8th. Medical graduates should be required to have their special fitness determined by the tests of proposition five.

9th. That failure to fulfill the requirements in any one branch should be sufficient to disqualify candidate to receive a certificate of capacity.

10th. In case a candidate fails in the practical tests in either of the two general departments of dentistry, he shall not be considered as not qualified.

11th. In considering the question of the examination of candidates for the special degree of dental surgery by a Board of Examiners established by law in each State, instead of by the present mode of examination by faculties, we submit as our belief, that the power to grant degrees must at length become vested in Boards of Examiners created for that purpose.

12th. The State Boards should have power to revoke for cause a certificate of qualification.

The Association reserved action upon the foregoing propositions until their next sitting and adjourned to meet the following morning at 8 a. m.

Meeting called to order by President Waters Aug. 6th, 1890, 8 a. m., roll was called, minutes of previous meeting read and approved. Discussion upon the propositions made by the committee on formulating principles of dental laws and which were made at the last session, was resumed and upon motion of Dr. Taft, the propositions were considered and acted upon in serial order. Proposition 1st was adopted without change. For proposition II Dr. Koch proposed as a substitute the following: We recommend that these Boards be officially created by the constituted appointing power of the various States, who shall select the appointees from a number of names presented to them by their respective State societies, and that each State society shall, at its annual meeting, place in nomination two names for each appointment to be made, so ordered. Dr. Taft moved as an amendment to proposition III that the words "five years" be substituted for the words "ten years." Proposition as thus amended was adopted. Propositions IV, V, VI, VII, VIII, IX, X, XI and XII were adopted, and upon motion of Dr. Wasson the report of the committee on formulating of vital principles of dental laws was then as a whole adopted. Dr. Koch from the committee on dental colleges presented the following report:

*Whereas*, complaints have been made to the National Association of Dental Examiners that recent graduates from the New York College of Dentistry, the dental department of the University of Pennsylvania, the Philadelphia Dental College, the Baltimore Dental College and the dental department of the University of Iowa, have been found not to possess sufficient knowledge or practical ability, by the State Boards of Massachusetts, Minnesota, New Jersey and Colorado, to permit them to practice in those States, it is ordered that the secretary inform the National Association of Dental Faculties of these complaints and urge upon all colleges to be more careful in the admission of students and the final examinations for graduation. Colleges, whose graduates may be discredited by State Boards hereafter by reason of their insufficient knowledge or ability, will be subject to have the endorsement, heretofore granted them by this Association, withdrawn.

Upon motion of Dr. Taft, the foregoing report was accepted and adopted. The Association then adjourned until half past four o'clock same day.

The Association was called to order Aug. 6th, 1890, at 4:30 p. m., President Waters being in the chair. The roll was called and minutes of previous meeting read and approved. On motion of Dr. Wasson it was

*Resolved*, That the secretary be instructed to prepare the minutes of the proceedings of this Association for publication and that he make arrangements with some one of the dental journals for their publication; also for a sufficient number of reprints to furnish each of the dental colleges and State boards of dental examiners with a copy of the same, so ordered. It was moved by Dr. Koch that a committee consisting of Drs. Taft, Wasson and Martindale, be appointed with instructions to memorialize Congress, in the name of this Association, to pass an act to regulate the practice of dentistry in the District of Columbia, so ordered. A letter was then read by the secretary from Dr. Jas. Truman, of Philadelphia, requesting the National Association of Dental Examiners to place him (as a representative of the dental department of the University of Pennsylvania) in possession of such facts as are obtainable bearing upon the charges of incompetency made against one of the recent graduates of said college, notice of which had that morning been conveyed to the National Association of Dental Faculties by the National Association of Dental Examiners. Dr. Jack presented the following:

*Resolved*, That Dr. Truman's letter, concerning the imputation against the University of Pennsylvania (dental depart.) contained in our communication to the National Association of Dental Faculties, be filed and that the secretary be instructed to furnish the representative of the University of Pennsylvania all the facts, in our possession, connected with our action. Resolution was adopted.

Dr. William P. Church, of Providence, R. I., a member of the Rhode Island Board of Registration in Dentistry, being present, having his credentials for admission and being desirous of having his Board represented in the Association of Dental Examiners, was duly admitted to the privileges of the Association and his State admitted to membership. The election of officers was then called for, was conducted according to the requirements of Art. VI of the constitution and resulted in the election of President, Dr. Chas. R. E. Koch, of Chicago; Vice-President, Dr. L. C. Wasson, Topeka, Kan.; Sec'y and Treas., Dr. J. H. Martindale, Minneapolis, Minn. The new officers were then inducted into their offices, and President Koch appointed as the committee on observation of dental colleges, Drs. Jack, Waters, Hughes, Church and Martindale. Upon motion of Dr. Waters it was ordered that a committee consisting of Drs. Taft, Waters and Martindale be appointed to procure, audit and place in proper custody the properties and effects of the National Association of Dental Examiners, now in the hands of Dr. F. A. Levy. Dr. Jack presented the following:

*Resolved*, That the sec'y and treas. be authorized to assess and collect of the secretary of each State Board of Dental Examiners a sufficient sum (within the constitutional limit) to meet the probable expense of the year 1890, in each case crediting them for this assessment, those States which have paid the assessment for the year 1889. Upon motion of Dr. Taft it was ordered that a committee of three be appointed by the president to arrange for the time and place of next meeting of this Association, that this committee be empowered with full authority to act for the Association and that they are requested to arrange for a meeting of the Asssociation at a different time from the meeting of the American Dental Association and at whatever place they deem best. President Koch appointed as such committee Drs. Taft, Levy and Garber. The Association then adjourned subject to the call of the President. J. H. Martindale,

Sec'y and Treas., Minneapolis, Minn.

